# Optical Coherence Tomography Guided Laser Cochleostomy: Towards the Accuracy on Tens of Micrometer Scale

**DOI:** 10.1155/2014/251814

**Published:** 2014-09-11

**Authors:** Yaokun Zhang, Tom Pfeiffer, Marcel Weller, Wolfgang Wieser, Robert Huber, Jörg Raczkowsky, Jörg Schipper, Heinz Wörn, Thomas Klenzner

**Affiliations:** ^1^Institute for Anthropomatics and Robotics (IAR)-Intelligent Process Control and Robotics (IPR), KIT, Engler-Bunte-Ring 8, 76131 Karlsruhe, Germany; ^2^Ludwig-Maximilians-University, Oettingenstraße 67, 80538 Munich, Germany; ^3^Department of Otorhinolaryngology, Düsseldorf University Hospital, Moorenstraße 5, 40225 Düsseldorf, Germany; ^4^Institute for Biomedical Optics, University of Lübeck, Gebäude 81, Raum R 67, Peter-Monnik-Weg 4, 23562 Lübeck, Germany

## Abstract

Lasers have been proven to be precise tools for bone ablation. Applying no mechanical stress to the patient, they are potentially very suitable for microsurgery on fragile structures such as the inner ear. However, it remains challenging to control the laser-bone ablation without injuring embedded soft tissue. In this work, we demonstrate a closed-loop control of a short-pulsed CO_2_ laser to perform laser cochleostomy under the monitoring of an optical coherence tomography (OCT) system. A foresighted detection of the bone-endosteum-perilymph boundary several hundred micrometers before its exposure has been realized. Position and duration of the laser pulses are planned based on the residual bone thickness distribution. OCT itself is also used as a highly accurate tracking system for motion compensation between the target area and the optics. During* ex vivo* experimental evaluation on fresh porcine cochleae, the ablation process terminated automatically when the thickness of the residual tissue layer uniformly reached a predefined value. The shape of the resulting channel bottom converged to the natural curvature of the endosteal layer without injuring the critical structure. Preliminary measurements in OCT scans indicated that the mean absolute accuracy of the shape approximation was only around 20 *μ*m.

## 1. Introduction

The inner ear is embedded in the temporal bone as part of the human skull. Future inner ear surgery will require a highly precise and most atraumatic surgical approach to the human cochlea with the organ of hearing. This is mandatory to give the possibility for future treatment options for diseases of the inner ear, for example, to place devices like drug delivery systems, electrodes, or optical fibers with preservation of existing inner ear function such as hearing or balance [1–3]. As an important surgical step, cochleostomy provides the surgical approach to the human cochlea when a round window approach is inconvenient or impossible, enabling the implantation of intracochlea devices. Preservation of existing inner ear function such as hearing or balance during this process is required.

**Figure 1:**
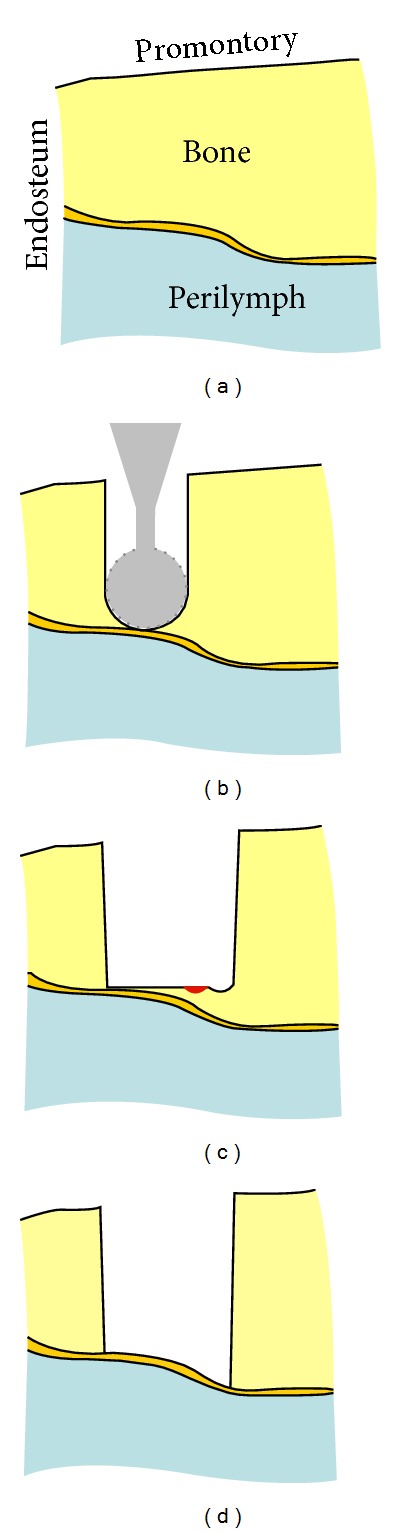
((a)-(b)) Conventional cochleostomy: exposing the endosteum of the scala tympani with a small burr (diameter 0.6 mm); ((c)-(d)) conception of laser cochleostomy: the bony shell of the cochlea is ablated pulse by pulse using a short-pulsed CO_2_ laser and the shape of the endosteum can be approximated more precisely. The red spot denotes the tiny tissue volume being ablated [18].

In clinical routine, a cochleostomy is manually drilled by the surgeon with diamond burrs (Figures 1(a) and 1(b)) to create an artificial channel for the implant on the bony shell of the cochlea. During this process, the cochlear endosteum should remain intact, preventing the bone-debris produced during the drilling process or blood from entering the scala tympani and meanwhile avoiding the leakage of the perilymph. Otherwise, the residual function of the cochlea will be damaged.

Due to the small diameter of the cochleostomy (approx. 1 mm) and the thickness of the fragile endosteal layer (<50 *μ*m), the required reproducible accuracy of the drilling process reaches the limit of human capabilities. The achievable precision of the manually performed cochleostomy is mainly dependent on the skills and experiences of the surgeon. Although operated with great care, the burr frequently tears or perforates the cochlear endosteum despite the surgeons' best efforts [4]. According to the review study of Incerti et al. [5], an insertion of electrodes into the cochlea is possible with preservation of residual hearing. Nonetheless the patients treated for electric acoustic stimulation on the same ear suffered from a postoperative threshold shift up to 30 dB in the deep frequencies. A computer-assisted microsurgery system is therefore desired to support the surgeons to ensure a reproducible precision during this highly demanding surgery.

For this purpose, robotic systems are common choices, using either a highly precise hexapod [6, 7] or a standard robot arm [8, 9]. Based on preoperative planning in CT scans, the robot is navigated to perform the drilling without violating critical structures like the facial nerve and the chorda tympani until reaching the stop point located on the endosteum. Unfortunately, the accuracy of the stop point in the planning data is limited by the resolution of the CT scan, which is about 0.1–0.25 mm in clinical routine and is insufficient regarding the thickness of the endosteum that is only several tens of micros. Unavoidable intraoperative registration and navigation errors further worsen the situation. Du et al. [10] and Brett et al. [11] therefore developed autonomous surgical robotic systems with real time haptic feedback. The critical structure is discriminated by analyzing the force and torque measured from the tip of the drill bit. The drilling will be ceased when a significant change in force and torque occurs, indicating that the endosteum is reached.

An inherent shortcoming of mechanical drilling is that the resulting channel bottom has the same shape as the burr that is convex in the direction of the drilling (Figure 1(b)). But, the cochlea is convex towards outside (see also Figure 3), that is, in the opposite direction of the drilling. As a result, while the drill bit already touches the endosteum in the middle part of the channel, some residual bone tissue still remains near the wall of the cochleostomy. In such a case, it is a challenging task to expose a sufficiently large area of the endosteum that matches the diameter of the implant without damaging the already exposed thin membrane in the middle. Moreover, mechanical drilling is always accompanied by high frequency vibration of the surrounding tissue, which might bring additional acoustic trauma to the cochlea.

Researches throughout the last decade revealed the feasibility of using a short-pulsed CO_2_ laser for hard tissue ablation [12–15] and more particularly for the inner ear surgery [16, 17]. Applying cooling water spray, the CO_2_ laser is able to achieve clean cuts on bone with no significant thermal injury to the surrounding tissue [12–15]. Compared to conventional surgical burrs, lasers allow contactless removal of the bone tissue in the absence of any mechanical stress to the fragile structures, providing more safety to the patient. The tiny tissue volume ablated by each single pulse enables a precise control of the channel geometry, which makes it possible to approach the natural curvature of the critical structure (Figures 1(c) and 1(d)). Laser ablation also generates much less bone-debris, reducing the risk of inflammatory tissue reaction of the inner ear and consecutive loss of function. In other words, lasers provide an excellent solution to the shortcomings of the mechanical drilling-based systems stated above.

However, a key question of using a laser to create the cochleostomy remains unsolved: how can the position of the bone-endosteum-perilymph boundary be detected during the process, so that the laser-bone ablation can be guided without injuring the critical structure?

In the past years, efforts have been made to solve this issue. The most popular choice is to discriminate the tissue type at the bottom of the laser-ablated incision. As soon as a transition from hard tissue to soft tissue is detected, the ablation process will be terminated. The tissue type differentiation can be achieved either by monitoring the ablation induced process emissions such as the plasma [20, 21] and noises [22–24], as well as both of them [25, 26], or by analyzing the optical properties of the tissue [18, 27–31]. A significant drawback of these approaches is that they can detect the tissue transition only after the tissue boundary has been penetrated, so that an injury to the critical structure is almost unavoidable.

Recently, several research groups have been using optical coherence tomography (OCT) to control the laser ablation [19, 32, 33]. These approaches mainly focus on measuring the position of the target tissue surface in the OCT scans such that the current ablation depth can be determined online with high accuracy on the micrometer scale. The ablation will be terminated as soon as the planned ablation depth is reached. However, these approaches will face the same problem as the robotic systems without haptic feedback [6–9] analyzed above, where the achievable accuracy is limited by the preoperative planning and intraoperative navigation.

Known as “ultrasonography with light,” the most valuable feature of OCT is that it can provide cross-sectional images of the internal structures beneath the target tissue surface with a high resolution on the micrometer scale [34, 35]. Therefore, OCT is potentially able to detect the position of the subsurface critical structure before its exposure. Instead of only monitoring the ablation depth, the laser pulses can be guided according to the thickness of the residual bone layer above the critical structure. In this paper, we will propose a closed-loop control of laser cochleostomy under the monitoring of OCT and report on preliminary experiments of OCT guided laser cochleostomy.

## 2. Materials and Methods

### 2.1. System Setup

An in-house built swept source OCT system consisting of a 54 kHz FDML laser [36] with a 104 nm sweep range at a center wavelength of 1314 nm was used to acquire the OCT scans. Its Rayleigh range was 2.0 mm. The axial and lateral resolutions were measured to be 18 *μ*m and 35 *μ*m, respectively. The cochleostomy was performed with a short-pulsed CO_2_ laser (wavelength 10.6 *μ*m, spot diameter 200 *μ*m, TEM_00_, and Rayleigh range 2.4 mm) with pulse durations tunable from 20 *μ*s to 100 *μ*s, corresponding to energies ranging from 4.2 mJ to 28.5 mJ per pulse. The OCT system and CO_2_ laser were equipped with separate scanning optics, which enabled simultaneous imaging and ablation of the target tissue surface. The angular resolution and repeatability of the scanning optics are <15 *μ*rad (OCT, Thorlabs GVS002) and <20 *μ*rad (CO_2_ laser, ARGES Colibri 11), respectively, corresponding to a spatial accuracy of* circa* 2-3 *μ*m within their working spaces.

**Figure 2:**
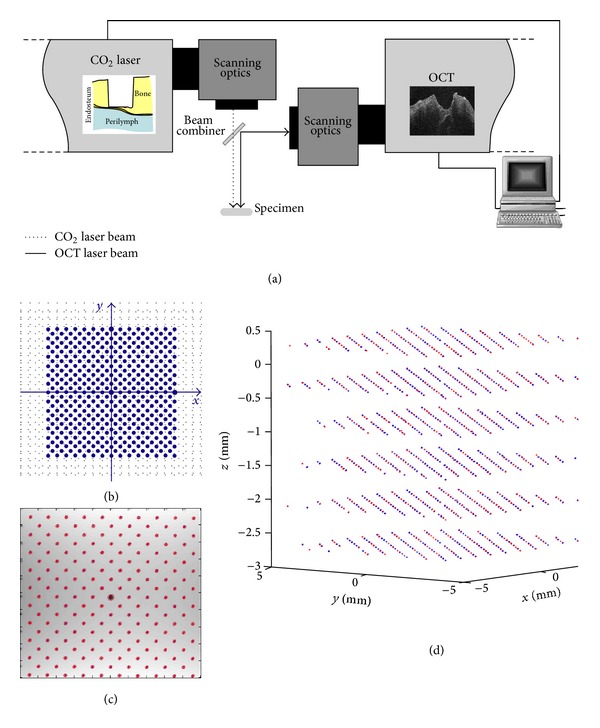
(a) Schematic diagram of the coaxial setup of the OCT system and the CO_2_ laser with overlapping working spaces; (b) two-dimensional calibration pattern defined in the CO_2_ laser coordinate system and (c) the corresponding points detected in the OCT coordinate system; (d) the corresponding point pairs filling the whole working space.

A coaxial setup of both systems (Figure 2(a)) was constructed using a dichroic germanium mirror with high reflectivity coating for the wavelengths of the OCT, so that the working spaces of the OCT and CO_2_ laser were overlapping. To create a three-dimensional mapping between both scanning optics, a calibration pattern was defined in the CO_2_ laser coordinate system (Figure 2(b)) and ablated on the surface of a flat acrylic plate. The position of each point was detected in a subsequent 3D OCT scan (Figure 2(c)), resulting in corresponding point pairs in both systems. This procedure was repeated at predefined axial positions that are equidistant along the optical axis, using a new acrylic plate each time. A calibration point cloud covering the whole working space was then obtained (Figure 2(d)). The mapping from OCT to CO_2_ laser (*x*, *y*, *z*) = *f*(*u*, *v*, *w*) was determined by performing tricubic B-spline fitting to these points.

Given a point (*x*, *y*, *z*) in the CO_2_ laser coordinate system and its corresponding point (*u*, *v*, *w*) in the OCT, the mapping error was defined as |(*x*, *y*, *z*) − *f*(*u*, *v*, *w*)|. We further defined an evaluation pattern consisting of the centers of all small squares (rotated for 45°) in the calibration pattern (Figure 2(b)). Using this pattern, the above ablation-detection procedure was repeated at the middle points of the equidistant axial positions that were used for the calibration. Thus, an evaluation point cloud containing points farthermost from the calibration points was obtained. The mean absolute mapping errors among the calibration points, among the evaluation points, and among all points together were 12.1 *μ*m, 29.3 *μ*m, and 19.6 *μ*m, respectively.

Such a calibration procedure has to be done only once during system setup and a recalibration is not necessary as long as both scanning optics and the OCT reference arm length remain fixed.

### 2.2. Feasibility Study

**Figure 3:**
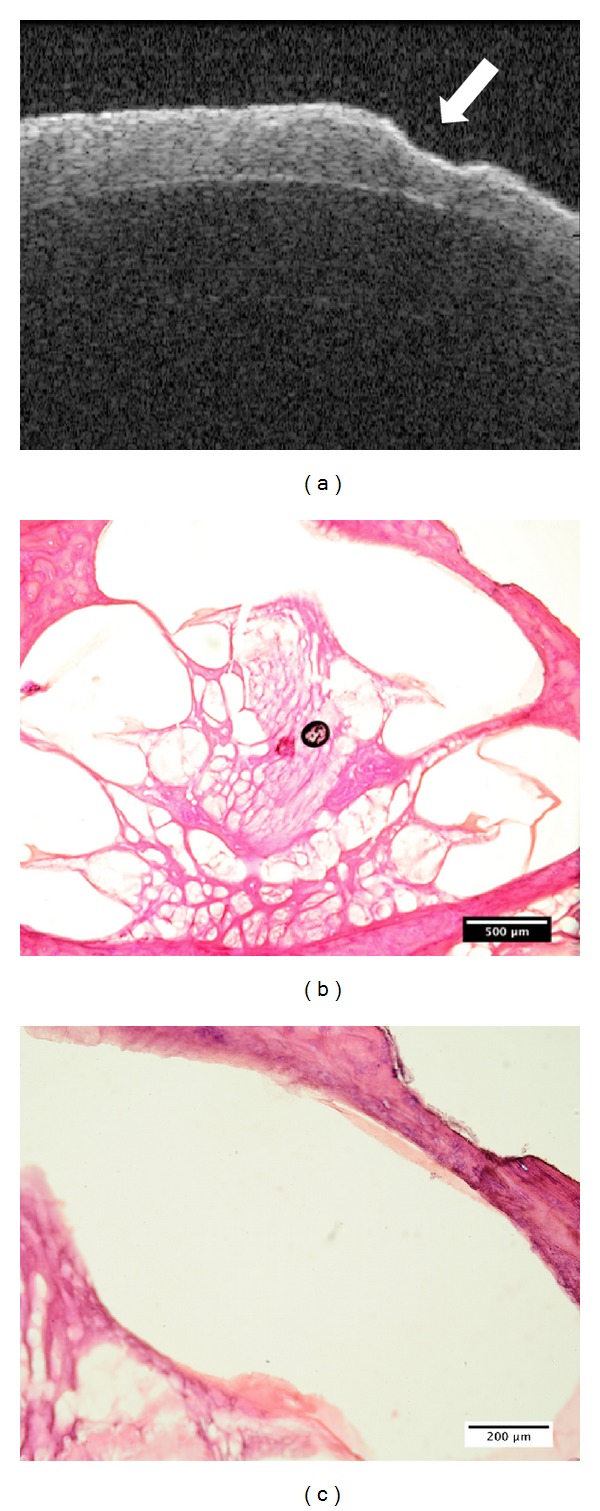
Feasibility study: (a) an OCT image of a fresh porcine cochlea with a laser-ablated crater on the surface (arrow). The interface between the bony shell of the cochlea, endosteum, and the perilymph-filled scala is clearly visible; ((b)-(c)) the corresponding histology under (b) 4x and (c) 10x magnification.

As the first step, a feasibility study was conducted by acquiring OCT scans on diverse fresh porcine cochleae isolated from cadavers and comparing them to histological sections (Figure 3). It can be observed that the interface between the bony shell of the cochlea, endosteum, and the perilymph-filled scala is clearly visible in the OCT. These results evidence the possibility to detect the position of the critical structure in the OCT scans before the endosteal layer is reached.

By analyzing the OCT scans of a wedge-shaped bovine compact bone specimen, it could further be estimated that the imaging depth of OCT penetrating into compact bone tissue is about half a millimeter under laboratory condition. Compared to the ablation depth of a single CO_2_ laser pulse ranging from 20 *μ*m to 100 *μ*m, the critical structure will be visible at least 4-5 ablation rounds before its exposure, so that the laser parameters such as pulse positions and pulse durations can be planned in advance to avoid injuring the fragile endosteum. Therefore, OCT is a very promising candidate for guiding the laser pulses during the laser cochleostomy.

### 2.3. OCT Guided Laser Cochleostomy

**Figure 4:**
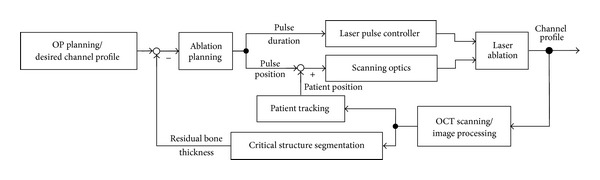
Control loop scheme of the OCT guided laser cochleostomy.

The control loop of the laser cochleostomy under the monitoring of OCT is designed as follows (Figure 4): the laser ablation and OCT scanning are performed alternately. After each round of ablation, a three-dimensional OCT volume scan of the cochleostomy is acquired. If the position of the bone-endosteum-perilymph boundary could be detected after proper image processing, the residual bone thickness above this critical structure can be calculated. Based on the obtained bone thickness distribution, the pulse positions and pulse durations for the next round of laser ablation are planned by a computer algorithm. After the compensation of potential relative displacement between the patient and the laser optics, the ablation parameters are transmitted to the corresponding control modules and the ablation pattern is executed. By repeating this procedure until the critical structure is reached, the desired endosteum preserving cochleostomy can be achieved.

### 2.4. Image Quality Enhancement

**Figure 5:**
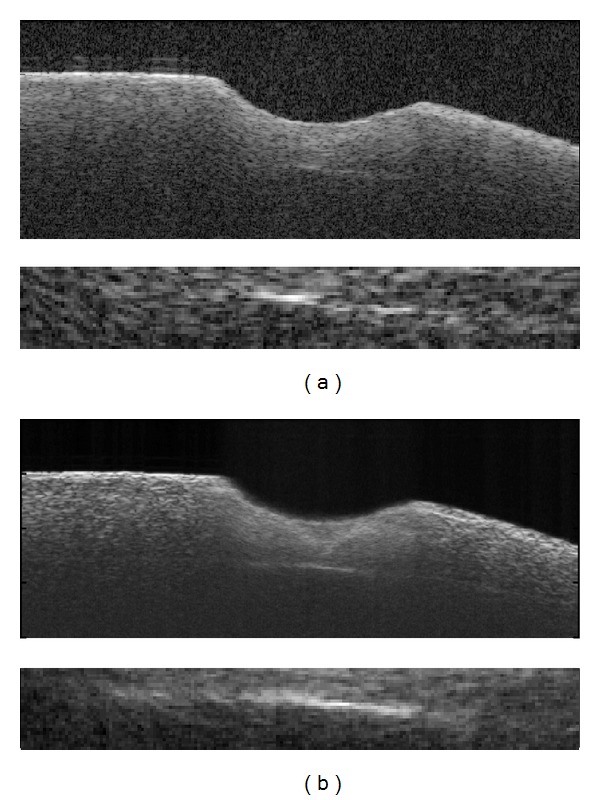
Effect of image quality enhancement: (a) original OCT image of a cochleostomy on a fresh porcine cochlea (above) and the zoomed view of the bone-endosteum-perilymph boundary (bottom); (b) the enhanced image after applying history compounding and light attenuation compensation techniques.

Obviously, the most crucial step in the control loop is the detection of the bone-endosteum-perilymph boundary. Unfortunately, lying beneath highly scattering bone tissue, the small signal coming from this critical structure is drowned out by multiple scattering. The speckle noise, which is inherent to OCT, further degrades the image quality. Moreover, the full sensitivity of the OCT system cannot be used due to its limited dynamic range and the presence of specular reflexes. As a result, the critical structure appears to be rather weak in the OCT (Figure 5(a)) and its detection is difficult.

Therefore, image quality enhancement must be applied before proceeding. We developed a new speckle averaging technique called “History Compounding” [37] and further applied the light attenuation compensation method proposed by Girard et al. [38] to increase the contrast of the structures deep beneath the bone surface. The combination of these techniques significantly improved the image quality of the OCT scans and the bone-endosteum-perilymph boundary became much clearer in comparison to the original one (Figure 5(b)).

### 2.5. Segmentation and Ablation Planning

**Figure 6:**
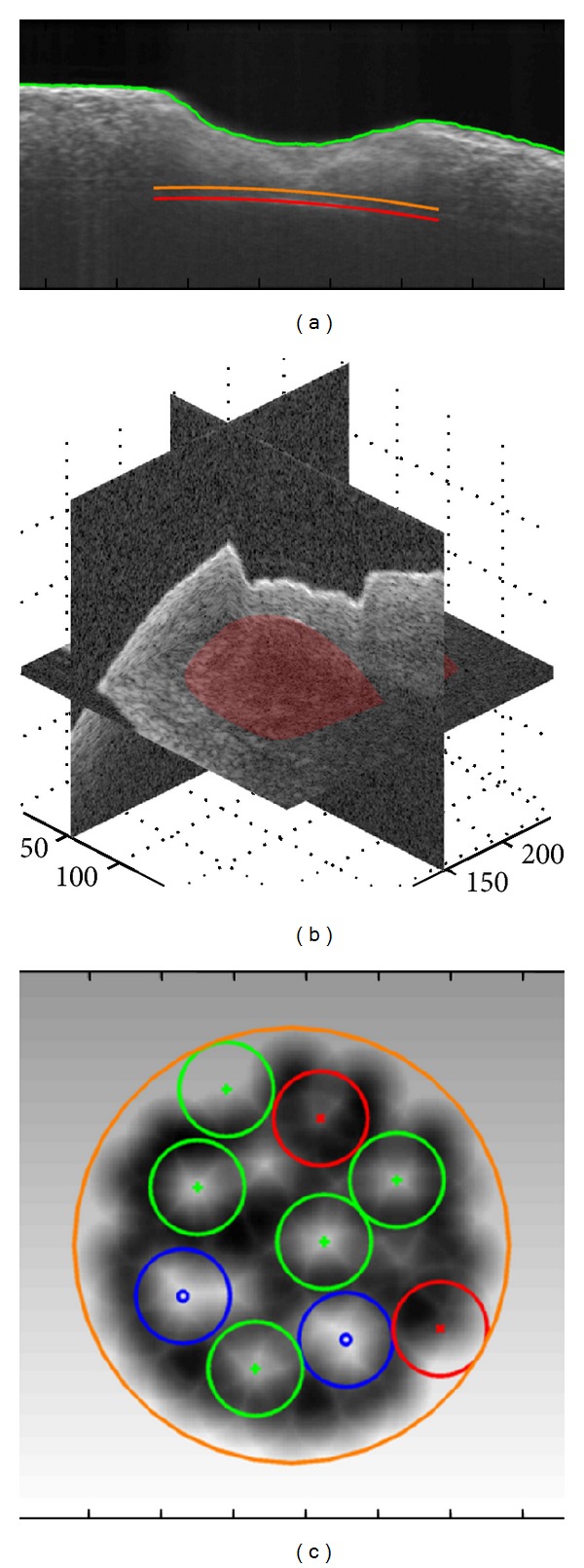
(a) The position of the bone surface (green), segmented bone-endosteum-perilymph boundary (red), and the user defined “stop surface” of the laser ablation (orange); (b) reconstructed 3D model of the critical structure; (c) ablation pattern planned according to the residual bone thickness map, where the blue, green, and red pulses are corresponding to long, middle, and short pulses; the gray scale in the background denotes the local residual bone thickness (lighter color for larger thickness).

The segmentation of such a sharp structure shown in Figure 5(b) is straightforward using gradient-based edge detection and model-based edge linking in each single OCT frame (Figure 6(a)). A bicubic B-spline fitting is performed to all candidate edge points in the whole three-dimensional OCT volume, taking the full use of information from neighboring frames and resulting in a smooth 3D model of the critical structure (Figure 6(b)). The user is further allowed to define a “stop surface” (Figure 6(a)) parallel to the detected bone-endosteum-perilymph boundary and the distance between them can be chosen arbitrarily.

Owing to the significantly different refractive indices of bone tissue and air, the most superficial air-bone interface always has very high contrast in OCT images. It can therefore be detected using simple thresholding and reconstructed by three-dimensional morphological operation-based smoothing (Figure 6(a)). The residual bone thickness distribution above the “stop surface” can be derived easily. Pulse positions and pulse durations are planned accordingly. The basic strategy is to apply the next pulse to the position with the maximal residual bone thickness where no pulses have been planned yet. The pulse duration is chosen quasi proportional to the thickness of the local bone layer. The ablation pattern for the next round of laser ablation (Figure 6(c)) is determined by repeating this procedure until no more pulses can be appended.

### 2.6. Patient Tracking

**Figure 7:**
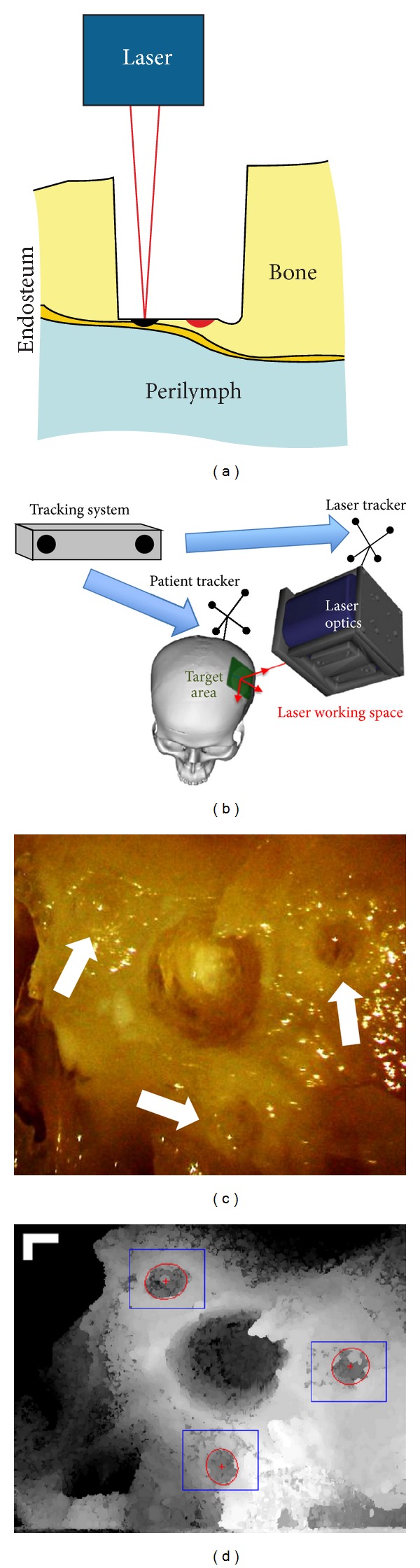
(a) Erroneously applied laser pulse (black) due to tiny relative displacement between target area and laser optics and the originally planned pulse position (red); (b) illustration of a typical setup using conventional tracking system; ((c)-(d)) OCT as highly accurate optical tracking system: (c) artificial landmarks surrounding the cochleostomy and (d) the corresponding top view in three-dimensional OCT scan, bar = 250 *μ*m [19].

Physical contacts to the patient, to the operation table, or to the laser optics due to incaution can cause relative displacements between the target area and the laser working space. As the diameter of the CO_2_ laser pulses is approximately 200 *μ*m, even tiny displacements less than 100 *μ*m can make the best ablation planning pointless. In the worst case, pulses shot to wrong positions can damage the endosteum instantly if parts of it are already exposed (Figure 7(a)).

Such displacements must be detected and taken into account before passing the planned pulse positions to the scanning optics of the CO_2_ laser. The gold standard for such a case is attaching special trackers to the patient as well as the laser optics and monitoring their positions using either optical or electromagnetic tracking systems, as illustrated in Figure 7(b). Similar setups are widely used by many research groups to perform computer-assisted cochleostomy [6–10, 32]. However, most commercially available tracking systems can only provide an accuracy of several hundred micrometers for each single tracker, while what we need is an accurate measurement of the relative displacement between the laser working space and the target area. Due to the indirect tracking mechanism of the conventional setups, registration between the target area and the patient tracker as well as between the laser optics and the laser tracker is mandatory, resulting in a complicated transformation chain from the target area via the tracking system to the laser working space. The registration and tracking errors of each component along this chain are accumulated. The large distances between the involved components further magnify the rotational tracking errors of the trackers, resulting in additional inaccuracy. As a result, conventional tracking system-based setups are almost impossible to achieve a global tracking accuracy less than 100 *μ*m regarding the relative displacement between the target area and the laser working space, which is insufficient in our case.

Therefore, we proposed a mechanism of using OCT itself as a more accurate optical tracking system [39] by locating small laser-ablated landmarks surrounding the cochleostomy (Figures 7(c) and 7(d)). The position of the target area can thus be determined directly in the OCT working space, bypassing the complicated transformation chain stated above. Because the cochleostomy is located near the centroid of the landmark layout, the rotational tracking error will not be magnified either. For the evaluation of the tracking accuracy, a specimen was moved along a predefined test grid within the laser working space using a hexapod (accuracy: ±2 *μ*m), whose position was tracked in the OCT. The global tracking accuracy of the target area with respect to the laser working space was measured by comparing the tracking results with the actual displacements performed by the hexapod, which was only about 25 *μ*m (mean absolute error: 22.8 ± 14.9 *μ*m, root mean square: 27.2 *μ*m).

## 3. Results and Discussion

### 3.1. Results

By now, the control loop conceived in Figure 4 has been successfully realized. The complete workflow was experimentally evaluated by conducting the worldwide first OCT guided laser cochleostomy on porcine cochleae isolated from cadavers.

Three cochleostomies were performed. No preoperative planning was made and the cochleae were manually positioned and oriented in the laser working space. The position of the bone-endosteum-perilymph boundary was unknown while starting the ablation process and the achieved accuracy was completely dependent on the proposed workflow. Before each round of ablation, water spray was manually applied to the target area, so that the ablation induced thermal injury could be effectively reduced and no significant carbonization was observed in the resulting cochleostomy. Meanwhile, the water spray also prevented tissue dehydration that can severely disturb the OCT imaging of the critical structure.

**Figure 8:**
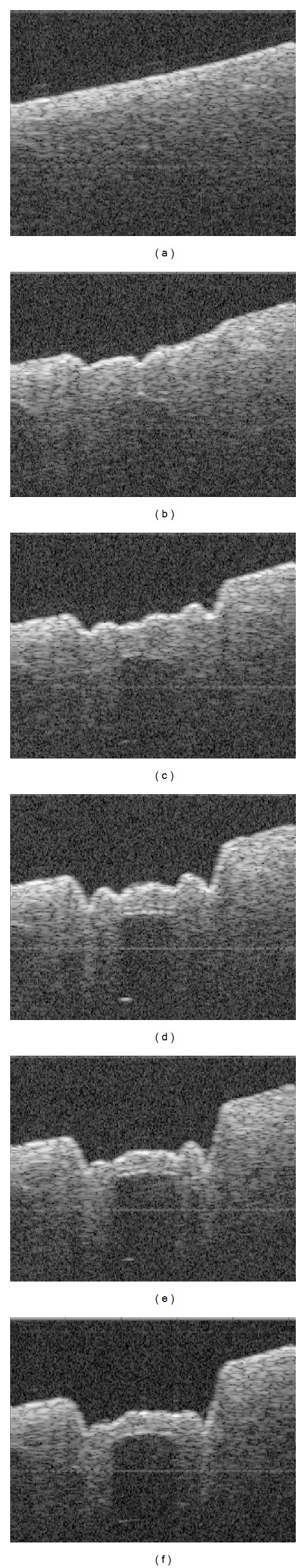
OCT frames passing through the center of the ablated cochleostomy showing the changing channel shape during the process of OCT guided laser cochleostomy on a fresh porcine cochlea.

Figure 8 shows the changing channel shape during one of the cochleostomies. At the beginning (Figures 8(a) and 8(b)), the bone-endosteum-perilymph boundary was still barely visible due to the relatively thick overlying bone layer. During this phase, the ablation was planned according to a virtual critical structure located at infinity and parallel to the original bone surface, resulting in a channel bottom approximately parallel to it (Figure 8(b)). With the increasing channel depth, the critical structure became gradually visible (Figure 8(c)). After applying the image quality enhancement and critical structure segmentation, the ablation was planned according to the bone thickness distribution measured online. As a result, the channel bottom began to incline clockwise and its shape converged to that of the endosteal layer step by step as expected (Figures 8(d)–8(f)).

**Figure 9:**
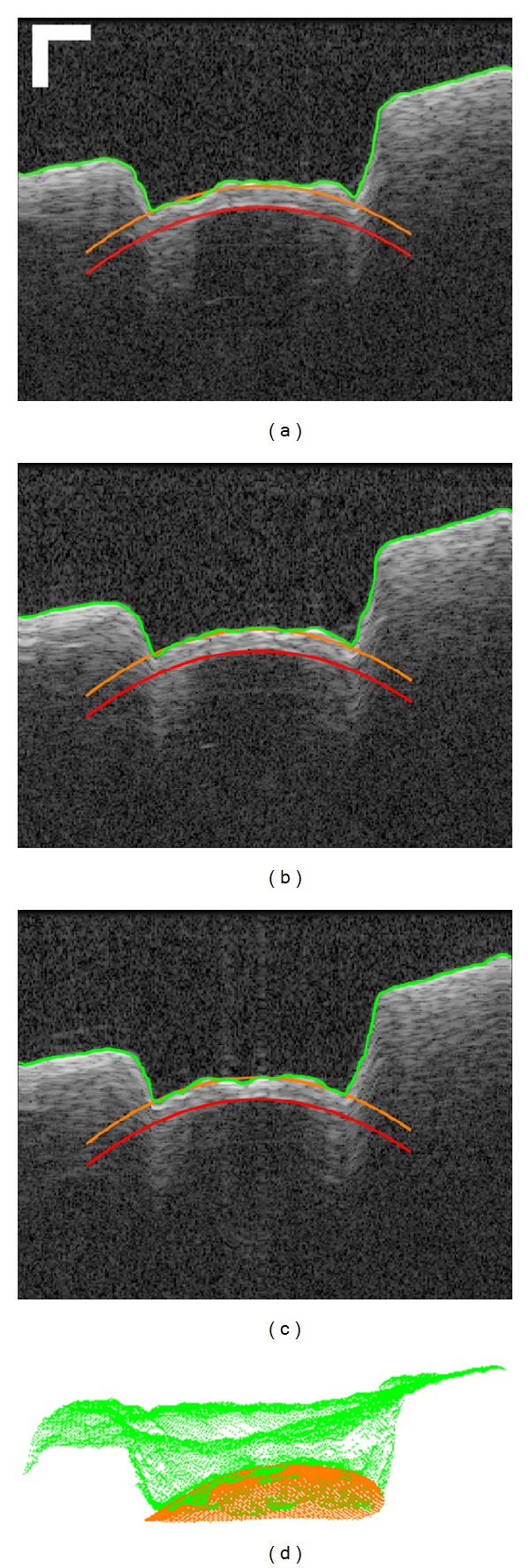
((a)–(c)) Example OCT B-scans acquired near the center of a resulting cochleostomy, showing the final shape of the ablated cochleostomy, bar = 250 *μ*m. (d) Comparison between the resulting channel bottom (green) and the three-dimensional “stop surface” (orange).

The target thickness of the residual layer was set to 100 *μ*m. The control loop quitted automatically when the user defined “stop surface” was reached all over the channel bottom. Preliminary investigation in postoperative OCT scans indicates that the shape of the resulting cochleostomy macroscopically matches the curvature of the cochlear endosteum (Figures 9(a)–9(c)).

Instead of only evaluating the ablation accuracy at a single point, a more strict evaluation comparing the whole channel bottom with the “stop surface” was performed (Figure 9(d)). According to the measurement in the postoperative OCT scan, the mean absolute errors between the resulting channel bottom and the three-dimensional “stop surface” were 16.43 ± 14.90 *μ*m, 19.62 ± 17.67 *μ*m, and 21.01 ± 21.39 *μ*m for the three cochleostomies, respectively. The corresponding maximal errors where the channel bottom penetrated the “stop surface” were 45.54 *μ*m, 38.36 *μ*m, and 46.73 *μ*m. An evaluation of the accuracy based on histological studies is still to be made.

### 3.2. Discussion

The preliminary result of the experimental evaluation reveals that, under the monitoring of the OCT, the laser ablation can be directly guided according to the residual bone thickness above the bone-endosteum-perilymph boundary measured online. In contrast to the control conceptions using other sensor technologies [18, 20–31], a foresighted detection of the critical structure before its exposure has been realized. Compared to the workgroups who have also been using OCT to guide the laser ablation [19, 32, 33], our approach does not only rely on measuring the bone surface but also take the full advantage of the tomographic information provided by the high-resolution imaging system.

A unique feature of our system is that the laser control module does not only control the laser on and laser off, but also optimize pulse positions and pulse durations according to the residual bone thickness distribution. To our knowledge, a uniform convergence of the resulting channel bottom to the shape of the critical structure has been demonstrated for the first time.

Reviewing the control loop, it can also be noticed that the workflow is independent of the type of the integrated ablating laser. The CO_2_ laser in the setup (Figure 2(a)) can be replaced by another kind of surgical lasers such as the commonly used Er:YAG laser.

On the other hand, our system is still an experimental setup and we have a long way to go before bringing it into real operation room. It can be observed that the resulting cochleostomy is not perfect and there exist in all three cochleostomies some positions where the channel bottom has penetrated the “stop surface” (Figure 9), indicating that a 100% protection of the endosteum has not been guaranteed yet. Meanwhile, the critical structure segmentation is currently semiautomatic. Due to the limited imaging depth of OCT, the critical structure is always invisible at the beginning (Figure 8(a)) or only a few pixels can be seen in the middle part (Figure 8(b)). The segmentation is impossible in the first case and often returns a wrong result in the later one. A manual correctness check of segmentation result is still mandatory. Further improvement of the ablation strategy and a more intelligent segmentation algorithm are therefore necessary.

Time consumption is another critical issue in the current system implementation. The OCT imaging, processing, and the CO_2_ laser control are done by three independent software packages and a manual data transfer between them is required. This has led to unnecessary overhead and allows human error to happen. Depending on the initial conditions including bone thickness and shape of the underlying endosteal layer, the OCT guided laser cochleostomy may cost up to more than one hour. Due to the manual data transfer, a real time tracking of the patient movements using the proposed tracking mechanism is also impossible in the current state. We are now working on speeding up the process by unifying the software packages and implementing GPU-based algorithms.

Further extensive systematic evaluations regarding the reliability, robustness, and repeatability of the system under different conditions are also essential.

## 4. Conclusions

In this work, we successfully solved a key problem hindering the clinical application of laser cochleostomy. Foresighted detection of the bone-endosteum-perilymph boundary in three-dimensional OCT volumes has been realized, enabling a residual bone thickness-based control mechanism of the laser ablation. An important step towards a standardized cochleostomy with reproducible ablation accuracy has been achieved. Future development of the OCT guided laser ablation system will provide the surgeon with a new intelligent microsurgical tool to perform the highly demanding surgical procedure in an easier but safer and more reliable way.
